# Is 3 cm the upper limit of stone size for effective steerable ureteroscopic renal stone evacuation?

**DOI:** 10.1007/s00345-026-06310-7

**Published:** 2026-02-27

**Authors:** Wei-Jen Chen, Feres Camargo Maluf, Hannah Jarvis, Zachary Burns, Vicente Elorrieta, Joseph Crivelli, Thomas Chi, Dean G. Assimos, Kyle D. Wood

**Affiliations:** 1 Department of Urology, University of Alabama at Birmingham, Birmingham, AL USA; 2 Department of Urology, Taipei Veterans General Hospital, Taipei, Taiwan

**Keywords:** Ureteroscopes, CVAC aspiration system, Stone-free, Nephrolithiasis

## Abstract

**Purpose:**

The CVAC^®^ Aspiration System is a novel device designed to actively evacuate stone fragments during ureteroscopy. While recent studies have validated its safety, the precise upper limit of stone size for this treatment is undefined. We aimed to determine the ideal size and volume thresholds using CVAC for steerable ureteroscopic renal evacuation (SURE) procedures.

**Methods:**

A retrospective, observational study of patients undergoing SURE procedures by a single surgeon between August 2023 and April 2025 was undertaken. Stone size and volume were quantified using preoperative CT imaging. The primary outcome was stone-free status, based on Endourological Society criteria for CT, or <5 mm for ultrasound/X-ray. Receiver Operating Characteristic (ROC) curve analyses were performed to estimate optimal cut-off values for stone size and volume.

**Results:**

Stones in 55 kidneys of 50 patients were removed with this approach. The majority had significant medical co-morbidities (82% ASA Class 3 or 4). The median stone size was 24.0 mm, and the overall stone-free rate (SFR) was 63.6%. For stones smaller than 2 cm, the SFR was 95.7%. ROC analysis identified a stone size of 30.9 mm (AUC 0.895) and a volume of 1501.1 mm³ (AUC 0.834) as predictive cut-offs for clearance. The volume threshold increased to 2264.6 mm³ with utilization of the CVAC 2.0 device. The overall complication rate was 12%, without any high-grade complications.

**Conclusions:**

The SURE procedure with the CVAC device may be effective for treating patients with stones up to 3 cm in size or volumes less than 2200 mm³. Well-designed, randomized prospective studies are needed to confirm these findings.

**Supplementary Information:**

The online version contains supplementary material available at 10.1007/s00345-026-06310-7.

## Introduction

Ureteroscopy is the most commonly performed stone removing procedure in the United States [1]. Several technologic advancements have been developed to facilitate ureteroscopic stone removal including the introduction of suction-based devices designed to actively evacuate stone fragments during and after lithotripsy. One such technology is the CVAC^®^ Aspiration System (Calyxo, Inc., Pleasanton, CA), which is used for Steerable Ureteroscopic Renal Evacuation (SURE) procedures. This device removes stones up to 2.5 mm in diameter. The ASPIRE trial (ASPiration to Improve Renal calculi removal Effectiveness), a multicenter, prospective, randomized noninferiority controlled trial, demonstrated that total stone clearance was significantly higher and residual stone volume significantly lower as compared to traditional ureteroscopy [2]. To date, there have been two generations of CVAC^®^ devices. The first-generation instrument (CVAC 1.0) consists of an irrigation and aspiration catheter allowing removal of stones ≤ 2.5 mm. The second-generation device (CVAC 2.0) consists of disposable digital flexible ureteroscope containing an aspiration channel allowing simultaneous laser lithotripsy and active suctioning of fragments ≤ 2.5 mm.

While recent studies have validated the safety of CVAC [3, 4], the upper limit of stone size amenable to CVAC treatment remains undefined. Therefore, we undertook a retrospective study in an attempt to define this limit.

## Materials and methods

### Study design and patient population

This retrospective, observational study was approved by the University of Alabama at Birmingham Institutional Review Board (IRB-300014501). Data on all cases where CVAC was employed between August 2023 and April 2025 were analyzed. All procedures were performed by a single fellowship trained endourologist (KW). The indications for SURE procedures included those based on standard patient guidelines [5, 6], as well as patients who had large renal stones but were ineligible for PCNL due to medical comorbidities including not being able to be treated off anti-coagulation or anti-platelet therapy, and excessive severe obesity where it was thought that prone PCNL would limit effective ventilation or supine PCNL extremely difficult to perform. Patients lacking a pre-operative CT imaging, those with renal anatomic abnormalities, nephrocalcinosis, or inadequate follow-up were excluded from analysis.

### Preoperative imaging and surgical technique

Preoperative stone size was measured by greatest length on preoperative non-contrast CT imaging (2 mm slice thickness). If multiple stones were present, cumulative stone diameters were quantified. Stone volume was calculated manually based on CT imaging using an ellipsoid formula (1/6π​×length×width×height). To ensure accuracy for branched stones volume calculation, the burden was manually compartmentalized into smaller segments, and the final volume was calculated as the sum of these individual components.

Preoperative ureteral stents were not routinely placed unless indicated for other reasons. All patients received SURE procedures under general anesthesia. A 12/14Fr ureteral access sheath (UAS) was used in all cases (Navigator, Boston Scientific, 36 cm in women, and 46 cm in men). Lasers utilized for stone fragmentation included a Thulium fiber laser (Soltive, Olympus) and a Holmium laser (MOSES 2.0, Lumenis). The Thulium fiber laser was used in the majority of cases while the Holmium laser was utilized only in situations where the Thulium fiber laser system was unavailable. Laser lithotripsy was initiated in dusting mode using the following parameters: 0.2 J and 120 Hz (long pulse) for the Thulium fiber laser, and 0.3 J and 80 Hz (Moses contact mode) for the Holmium laser. Settings were adjusted according to stone response. Stone basketing was based on the surgeon’s discretion. The selection of CVAC 1.0 or 2.0 devices was based on market availability rather than clinical factors. Indwelling ureteral stents (the majority with tether suture) were placed at the end of all cases and typically removed 3–5 days later. A reusable flexible ureteroscope (Karl Storz FLEX-XC digital) was used for initial inspection, and laser fragmentation. This approach was chosen as visualization was felt to be superior with the reusable ureteroscope. A CVAC device was used for subsequent fragment suctioning (CVAC 1.0 and 2.0) and further stone fragmentation and evacuation if still needed (CVAC 2.0).

### Post-operative follow-up

Follow-up imaging modalities and its timing varied and was based on surgeon discretion. All studies were undertaken within 3 months. For those with CT scan follow-up, stone-free was defined by Endourological Society criteria: Grade A (truly stone-free), Grade B (≤ 2 mm fragments), and Grade C (2 ~ ≤ 4 mm fragments). For those with ultrasound plus abdominal X-ray follow-up, stone-free was defined by no stone or less than 5 mm residual stones on ultrasonography and no visible stones on radiography.

Post-operative complications were defined as any adverse events occurring within 30 days after surgery that were related to the procedure.

### Statistical analysis

All data were collected using standardized forms and were analyzed using SPSS 31.0 software (SPSS Inc, Chicago, Ill). Categorical variables were reported as counts and percentages. Continuous variables were expressed as medians with interquartile ranges (IQRs). Comparisons were performed using the Fisher’s exact test for categorical variables, and the Mann-Whitney U test for continuous variables. Receiver Operating Characteristic (ROC) curves were performed to determine the cut-off values for stone size and volume regarding stone-free status. All statistical tests were two-tailed, and a *p* value less than 0.05 was considered statistically significant.

For patients undergoing bilateral procedures, stone characteristics and surgical outcomes were analyzed per renal unit. Demographic data, total operative time, stone composition, and complications were analyzed on a per-patient basis.

## Results

### Patient demographics and preoperative characteristics

A total of 66 patients who underwent CVAC procedures between August 2023 and April 2025 were initially screened. After excluding patients without preoperative CT scans (*n* = 5) or follow-up data (*n* = 6), as well as those with nephrocalcinosis (*n* = 2), renal malrotation (*n* = 2), and horseshoe kidney (*n* = 1), 50 patients accounting for 55 renal units were included in the final analysis. The demographic and preoperative data are summarized in Table 1. 48% of patients had positive pre-operative urine culture results and received culture directed antibiotic therapy prior to surgery. 54% of patients had multiple renal stones. The majority had medical co-morbidities; 82.0% ASA Class 3 or 4, 18% BMI > 40 kg/m^2^. Preoperative stone characteristics are outlined in Table S1 (see supplement). Median stone size was 24.0 mm (IQR 13.3–41.0), median stone volume 1320.8 mm³ (IQR 582.7–2270.8). More than half of the renal units contained lower pole stones.

### Intraoperative parameters

Intraoperative outcomes are detailed in Table S1 (see supplement). The mean total operative time was 115.02 ± 38.26 min. There was no statistically significant difference in operative time between cases with stone sizes > 3 cm and those ≤ 3 cm (117.71 ± 36.39 min vs. 112.54 ± 41.18 min; *p* = 0.64). This is likely due to the surgeon trying to limit operative time to less than 2 h. When the procedure was expected to take longer, a staged approach was undertaken which was done in 4 patients. Most procedures (92%) did not require the use of a basket reflecting CVAC’s suction efficiency. Stone analysis demonstrated calcium oxalate as the predominant component.

### Stone-free rates and influencing factors

The overall stone-free rate (SFR) was 63.6% (35/55 renal units) based on the criteria mentioned in Material and Methods session (Table 2). Table 3 compares the differences between the Stone-free and Not stone-free groups. The results show that mean stone size (16.0 vs. 42.8 mm, *p* < 0.001), stone volume (706.9 vs. 2385.8 mm³, *p* < 0.001), and stone number (1.0 vs. 3.5, *p* < 0.001) were significant factors influencing SFR, but not for maximal stone Hounsfield Unit on CT. Additionally, the presence of lower pole stones significantly reduced SFR (*p* = 0.007).

### ROC curve analysis and threshold determination

**Fig. 1:**
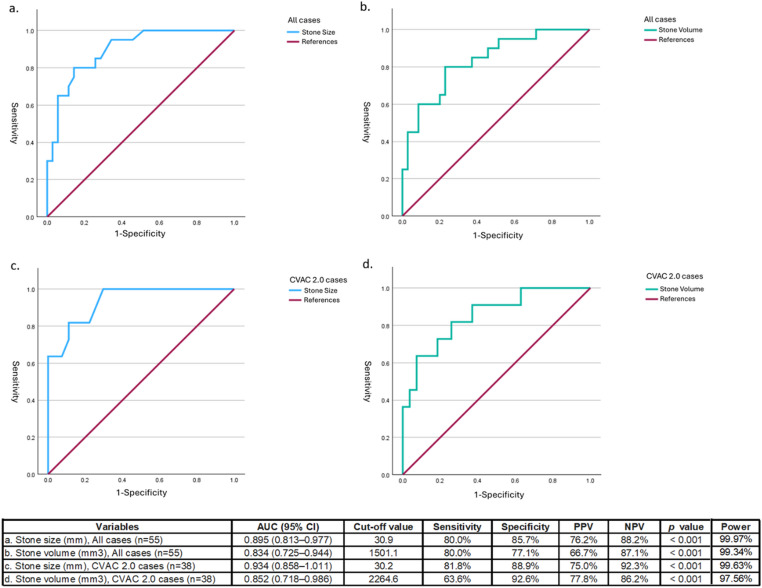
ROC curves of stone size and stone volume for predicting residual stones

In an effort to define the upper limit of stone size and volume for effective stone removal with CVAC, ROC curve analyses were performed (Fig. 1).

For all cases (*n* = 55), the AUC for stone size was 0.895 (95% CI: 0.813–0.977, *p* < 0.001). The statistically calculated optimal cut-off value was a stone size of 30.9 mm. This implies that when the stone size is less, there is a reasonable chance of effective stone removal with CVAC devices. Subgroup analysis for CVAC 2.0 cases (*n* = 38) showed even higher predictive power (AUC = 0.934), with an optimal cut-off value of stone size 30.15 mm. These data suggest the threshold for effective stone removal with a CVAC 2.0 device is approximately 3 cm.

Similar to the linear stone size measurements, stone volume also demonstrated strong predictive effective stone removal (AUC 0.834, 95% CI: 0.725–0.944, *p* < 0.001). The volumetric cut-off was identified at 1501.1 mm³. Subgroup analysis of CVAC 2.0 cases (*n* = 38) further revealed the predictive threshold for stone volume increased to 2264.6 mm³ (AUC 0.852). This suggests that while 3 cm remains the critical linear threshold, the CVAC 2.0 system is capable of effectively managing stone volume to around 2200mm^3^.

Although our sample size was limited, a post hoc power analysis confirmed that the study was adequately powered to detect these significant differences, supporting the reliability of these ROC-derived cutoffs (Fig. 1).

### Safety and complications

The overall complication rate was 12% (*n* = 6); all were Clavien-Dindo Grade I–II. One patient developed a perirenal fluid collection causing flank pain, which was managed conservatively; two patients developed postoperative urinary tract infections and were treated with oral antibiotics. One patient experienced an intraoperative Grade 2 ureteral injury [7] associated with ureteral access sheath (UAS) insertion, which was managed with an indwelling ureteral stent for six weeks. Following stent removal, diuretic renography performed 3 months and 6 months later demonstrated normal function and drainage of the involved renal unit. Two patients (4%) required subsequent hospitalization for treatment of sepsis; both with risk factors for this complication. One of these patients had a prior cerebral vascular accident with hemiparesis, and recurrent urinary tract infections history. She had received a 3-day course of culture specific preoperative intravenous antibiotics and was discharged on the day of surgery. She developed sepsis and acute kidney injury (AKI) on postoperative day (POD) 29. The second patient was severely obese (BMI 70 kg/m²) and had a preoperative urinary tract infection developed sepsis and AKI on POD 7. Both patients had uneventful recovery after sepsis management.

**Table 1:** Demographic data and pre-operative characteristics (50 patients)

Characteristics	
Age (years), mean ± SD	56.2 ± 14.7
Gender, n (%)	
Male	16 (32.0)
Female	34 (68.0)
BMI (kg/m²), median (Q1–Q3)	31.0 (25.0–37.5)
BMI ≥ 40 kg/m², n (%)	9 (18.0)
ASA Classification, n (%)	
Class 2	9 (18.0)
Class 3	38 (76.0)
Class 4	3 (6.0)
Surgical side, n (%)	
Left	29 (58.0)
Right	16 (32.0)
Bilateral	5 (10.0)
Ethnicity, n (%)	
White	36 (72.0)
Black or African American	9 (18.0)
Hispanic/Latino	3 (6.0)
Asian	2 (4.0)
Antiplatelet use, n (%)	9 (18.0)
Anticoagulant use, n (%)	7 (14.0)
Positive pre-op urine culture, n (%)	24 (48.0)
Preoperative stenting, n (%)	23 (46.0)
Stone numbers, median (Q1–Q3)	1.0 (1.0–4.0)
With multiple stones, n (%)	27 (54.0)
With ureteral stone, n (%)	9 (18.0)

*BMI* body mass index, *SD* standard deviation

**Table 2:** Stone-free criteria based on post-operative image modalities

Criteria regarded as stone-free	*n*(%)
CT Grade A (No residual fragments)	8 (14.5)
CT Grade B (Residual fragments ≤2 mm)	2 (3.6)
CT Grade C (Residual fragments 2 ~ ≤ 4 mm)	0 (0.0)
KUB and US reported no stone	17 (30.9)
US reported residual fragments ≤5 mm, and invisible on KUB	8 (14.5)
Total	35 (63.6)

Criteria regarded as not stone-free	n (%)
Residual fragments >4 mm on CT	7 (12.7)
Residual fragments >5 mm on US, and/or visible on KUB	13 (23.6)
Total	20 (36.4)

(55 renal units)

*KUB* Kidney, ureter, and bladder X-ray, *US* Ultrasound

**Table 3:** Operative outcome based on renal unit (*N* = 55)

	Stone-free (*N* = 35)	Not stone-free (*N* = 20)	*p* value
Stone size (mm), median (Q1–Q3)	16.0 (12.0–27.4)	42.8 (31.0–60.8)	< 0.001
Stone size ≤3 cm, n(%)	29 (82.9)	4 (20)	< 0.001
Stone volume (mm^3^), median (Q1–Q3)	706.9 (272.5–1477.5)	2385.8 (1539.4–5814.8)	< 0.001
Stone numbers, median (Q1–Q3)	1.0 (1.0–1.0)	3.5 (2.0–5.75)	< 0.001
Maximum stone HU, median (Q1–Q3)	940.0 (578.0–1422.7)	1024.0 (682.5–1452.5)	0.529
Containing lower pole stones, n(%)	17 (48.6)	17 (85)	0.007
Main component (*N* = 47): CaOx, n(%)	18 (66.7)	8 (40)	0.069

*HU* Hounsfield Unit, *CaOx* Calcium oxalate

## Discussion

This single-center retrospective study summarizes two years of experience with SURE procedure using CVAC systems. All procedures were performed by the same urologist. Many patients had significant medical co-morbidities; 82% with ASA Classification 3 or 4. The median stone size was 24 mm indicating that many of these patients would typically be managed with PCNL based on current AUA and EAU guidelines [5, 6]. However, there is emerging evidence suggesting that using new technology may allow this size threshold to be extended in ureteroscopy. A recent study by Liu et al. [8] reported a 91% SFR (no fragments > 2 mm) at 30 days postoperatively in 35 patients with 2.5–3.8 cm kidney stones treated using a flexible and navigable ureteral access sheath (FANS). Although CVAC is a different technology from FANS, it also combines active suction to improve SFR. In our series, through ROC curve analysis, we identified a stone size cut-off of 30.9 mm for ideal stone removal with this approach. This suggests that the CVAC system may offer a minimally invasive option for patients with “intermediate-size” stones between 2 and 3 cm.

Although our observed stone-free rate (63.6%) was lower than that reported [8] with FANS, this discrepancy may have been due to the patients harboring larger stones who were not thought to be good PCNL candidates. In fact, 30.9% (17/55) of our renal units had a stone size exceeding 3.8 cm; five units with stone size exceeding 6 cm. The stone-free rate in this subgroup was only 23.5% (4/17). However, with stone size less than the 30.9 cm threshold generated from the ROC analysis, the SFR was 88.2% (30/34). A threshold of 3 cm has been reported by others [9].

While using CVAC for treating patients with even larger stone size is a consideration, operative time may be prolonged and place the patient at risk for sepsis [10–12]. The mean operative time in this series was somewhat less than 2 h in an effort to reduce risks of sepsis. This is likely the reason for why operative times did not differ significantly between the ≤ 3 cm and > 3 cm groups. Staged procedures were undertaken in four patients with large renal stones. Among them, only one patient (4.4 cm stone size) achieved Grade A stone-free status after a second CVAC procedure, while the remaining three patients (all with stones size > 7 cm) were not rendered stone-free.

Some feel that stone volume is a better predictor of achieving a stone free result as compared to linear stone size. Ito et al. reported that this was especially true when the linear stone size exceeds 20 mm or when more than three stones are present [13, 14]. In our cohort, 21 of 22 patients with stones smaller than 2 cm achieved stone-free status. Therefore, with such a high stone free rate, we did not undertake ROC curve analysis for the subset of stones < 2 cm. When restricting the analysis to patients with stone size > 2 cm, stone size yielded an AUC of 0.771 (95% CI: 0.606–0.937, *p* < 0.001), which was marginally superior to stone volume (AUC 0.721, 95% CI: 0.544–0.897, *p* = 0.014). The analysis also confirmed optimal cut-off values of 30.9 mm for linear size and 2264.6 mm³ for volume (Figure not shown).

A recent meta-analysis by Geraghty et al. [15] assessed the prognostic value stone volume and linear stone size for predicting achievement of a stone free state with ureteroscopy. Based on the eight studies included in their review, traditional efficacy thresholds are typically 20 mm for stone size and 1000–1500 mm³ for volume; stone volume generally demonstrating a significantly higher AUC than size. In contrast, our findings suggest that the CVAC system may extend these therapeutic limits, potentially elevating the feasible threshold to approximately 3 cm and 2200 mm³. Although our analysis did not find stone volume to be statistically superior to stone size for predicting clearance, the difference in AUC was minimal. This discrepancy may be attributed to our relatively small sample size or the unique mechanism of the CVAC system. Unlike conventional ureteroscopy, where volume correlates with fragment retrieval difficulty, the active aspiration of CVAC may mitigate the “volume effect” to some degree, making the outcomes less dependent on total volume compared to diameter.

The CVAC 2.0 system utilizes a 12/14 Fr ureteral access sheath (UAS), a size that necessitates caution during placement to prevent trauma. Although guidelines do not formally mandate pre-stenting for 12/14 Fr sheaths, it is known to mitigate the risk of ureteral injury [16]. In our institution, preoperative stents were not routinely placed; however, 46% of patients presented with ureteral stents at time of referral. This introduces potential selection bias, as the requisite 12/14 Fr sheath is easier to deploy in pre-stented ureters. Notably, the only ureteral injury observed in our cohort occurred in a patient without a preoperative stent.

The high endoscope-sheath diameter ratio (RESD) of the 11.9Fr CVAC 2.0 within 12/14F UAS limits passive drainage from UAS. Maintaining intrarenal pressures below 40 cmH₂O [17] is challenging even with standard suction sheaths [18]; however, CVAC mitigates this risk through dedicated active aspiration. Our sepsis rate was not higher than previous reported for conventional flexible ureteroscopic stone removal [12, 19].

Mini-PCNL is another treatment for patients harboring 2–3 cm renal stones. It has been reported that compared to FANS in a propensity score-matched analysis, the stone free rate is higher and the need for re-intervention is lower. However, recovery time is longer [20]. We feel that based on our results with CVAC that a randomized prospective trial comparing CVAC to mini-PCNL for patients with 2–3 cm renal stones warrants consideration.

This study has limitations. It is a single-center retrospective study with a relatively small sample size. Thus, it should only be considered hypothesis generating. Heterogeneous postoperative imaging modalities may have introduced bias as we acknowledge that KUB/renal ultrasound is inferior to CT for detecting small residual fragments, particularly those smaller than 3 mm. In addition, combining the results from two different CVAC systems introduces another source of heterogeneity.

## Conclusions

Our study demonstrates the effectiveness and safety of the SURE treatment in managing renal stones. Our findings suggest that the CVAC system maintains a favorable safety profile and achieves high stone-free rates in patients with stone size up to 3 cm and stone volume up to 2200 mm³. Given the retrospective nature of the study, these findings should be considered hypothesis-generating. Well-designed, randomized prospective studies are needed to validate these findings.

## Supplementary Information

Supplementary Material 1

## Data Availability

The data supporting the findings of this study are derived from retrospective review of medical records and contain sensitive patient information. Therefore, the data are not publicly available. De-identified data may be made available from the corresponding author upon reasonable request and with approval from the institutional review board.

## References

[1] Monga M, Murphy M, Paranjpe R, Cutone B, Eisner B (2023) Prevalence of stone disease and procedure trends in the United States. Urology 176:63–68. 10.1016/j.urology.2023.03.040 37062518 10.1016/j.urology.2023.03.040

[2] Matlaga BR, Mueller TJ, Johnson B, Page J, Wolf JS Jr., Preminger GM et al (2025) A prospective, randomized, noninferiority study to evaluate the safety and effectiveness of steerable ureteroscopic renal evacuation compared with standard ureteroscopy: 30-day results of the ASPIRE study. J Endourol 39(1):10–18. 10.1089/end.2024.0602 39699687 10.1089/end.2024.0602

[3] Stern KL, Borgert BJ, Wolf JS Jr. (2023) Steerable ureteroscopic renal evacuation (SURE) for large renal stones: a multi-institutional center study. J Endourol 37(11):1179–1183. 10.1089/end.2023.0424 37639362 10.1089/end.2023.0424 PMC10663695

[4] Ballantyne CC, Foss HE, Cabo JJ, Turin D, Edmonds VS, Wymer KM et al (2025) First in-vivo multicenter experience with the novel CVAC 2.0 ureteroscope with simultaneous irrigation and aspiration functionality. World J Urol 43(1):645. 10.1007/s00345-025-05912-x 41162679 10.1007/s00345-025-05912-x PMC12572026

[5] Assimos D, Krambeck A, Miller NL, Monga M, Murad MH, Nelson CP et al (2016) Surgical Management of Stones: American Urological Association/Endourological Society Guideline, PART I. J Urol 196(4):1153–1160. 10.1016/j.juro.2016.05.090 27238616 10.1016/j.juro.2016.05.090

[6] Skolarikos A, Neisius HJA, Petřík A, Kamphuis GM, Davis NF, Somani B, Tailly T, Lardas M, Gambaro G, Sayer JA (2025) EAU Guidelines on Urolithiasis. https://uroweb.org/guidelines/urolithiasis. Accessed July 1st 2025

[7] Traxer O, Thomas A (2013) Prospective evaluation and classification of ureteral wall injuries resulting from insertion of a ureteral access sheath during retrograde intrarenal surgery. J Urol 189(2):580–584. 10.1016/j.juro.2012.08.197 22982421 10.1016/j.juro.2012.08.197

[8] Liu G, Zhang X, Xu Z, Li X (2025) A comparative study of flexible and navigable suction ureteral access sheath combined with single-use flexible ureteroscopes and percutaneous nephrolithotomy in the treatment of kidney stones > 2.5 cm: a single-center retrospective study. BMC Urol 25(1):226. 10.1186/s12894-025-01930-4 40898213 10.1186/s12894-025-01930-4 PMC12403407

[9] Daniel M, Klyde TA, Zeph Okeke DM, Hoenig A, Rai, Jared Swinoker (2025) PD01-06 real-world experience with cvac 2.0 for steerable ureteroscopic renal evacuation in a large, multi-site academic institution. J Urol 213(5S):e36. 10.1097/01.JU.0001109712.09934.41.06

[10] Whitehurst L, Pietropaolo A, Geraghty R, Kyriakides R, Somani BK (2020) Factors affecting operative time during ureteroscopy and stone treatment and its effect on outcomes: retrospective results over 6.5 years. Ther Adv Urol 12:1756287220934403. 10.1177/1756287220934403 32636935 10.1177/1756287220934403 PMC7313327

[11] Ozgor F, Sahan M, Cubuk A, Ortac M, Ayranci A, Sarilar O (2019) Factors affecting infectious complications following flexible ureterorenoscopy. Urolithiasis 47(5):481–486. 10.1007/s00240-018-1098-y 30448869 10.1007/s00240-018-1098-y

[12] Corrales M, Sierra A, Doizi S, Traxer O (2022) Risk of sepsis in retrograde intrarenal surgery: a systematic review of the literature. Eur Urol Open Sci 44:84–91. 10.1016/j.euros.2022.08.008 36071820 10.1016/j.euros.2022.08.008 PMC9442387

[13] Ito H, Kawahara T, Terao H, Ogawa T, Yao M, Kubota Y et al (2012) The most reliable preoperative assessment of renal stone burden as a predictor of stone-free status after flexible ureteroscopy with holmium laser lithotripsy: a single-center experience. Urology 80(3):524–528. 10.1016/j.urology.2012.04.001 22658621 10.1016/j.urology.2012.04.001

[14] Ito H, Kawahara T, Terao H, Ogawa T, Yao M, Kubota Y et al (2013) Utility and limitation of cumulative stone diameter in predicting urinary stone burden at flexible ureteroscopy with holmium laser lithotripsy: a single-center experience. PLoS ONE 8(6):e65060. 10.1371/journal.pone.0065060 23750229 10.1371/journal.pone.0065060 PMC3672212

[15] Geraghty R, Pietropaolo A, Tzelves L, Lombardo R, Jung H, Neisius A et al (2025) Which Measure of stone burden is the best predictor of interventional outcomes in urolithiasis: a systematic review and meta-analysis by the YAU urolithiasis working group and EAU urolithiasis guidelines panel. Eur Urol Open Sci 71:22–30. 10.1016/j.euros.2024.10.024 39651399 10.1016/j.euros.2024.10.024 PMC11625283

[16] Loftus CJ, Ganesan V, Traxer O, Schold JD, Noble M, Sivalingam S et al (2020) Ureteral wall injury with ureteral access sheaths: a randomized prospective trial. J Endourol 34(9):932–936. 10.1089/end.2018.0603 30526031 10.1089/end.2018.0603

[17] Sierra A, Corrales M, Kolvatzis M, Doizi S, Traxer O (2022) Real time intrarenal pressure control during flexible ureterorrenscopy using a vascular pressurewire: pilot study. J Clin Med 12(1). 10.3390/jcm12010147 10.3390/jcm12010147 PMC9821029 36614947

[18] Shi J, Huang T, Song B, Liu W, Cheng Y, Fang L (2024) The optimal ratio of endoscope-sheath diameter with negative-pressure ureteral access sheath: an in vitro research. World J Urol 42(1):122. 10.1007/s00345-024-04815-7 38453696 10.1007/s00345-024-04815-7

[19] Bloom J, Fox C, Fullerton S, Matthews G, Phillips J (2017) Sepsis after elective ureteroscopy. Can J Urol 24(5):9017–9023 28971790

[20] Cormio A, Castellani D, Yuen SK, Fong KY, Gadzhiev N, Kalathia J et al (2025) Head-to-head comparison of novel suction technologies in endourology: suction mini-PCNL versus flexible ureteroscopy using flexible and navigable suction ureteral access sheaths for renal stone management. An EAU-endourology, propensity score-matched analysis of 1372 patients. World J Urol 43(1):644. 10.1007/s00345-025-06024-2 41160140 10.1007/s00345-025-06024-2

