# Supplementary material for: Is 3 cm the upper limit of stone size for effective steerable ureteroscopic renal stone evacuation?
Source: World J Urol. 2026 Feb 27;44(1):208. doi: 10.1007/s00345-026-06310-7 (PMC12948787; doi:10.1007/s00345-026-06310-7)
Supplement: Supplementary file 1 — Supplementary Material 1 [file 345_2026_6310_MOESM1_ESM.docx]

**Is 3 cm the Upper Limit of Stone Size for Effective Steerable Ureteroscopic Renal Stone Evacuation?**

Wei-Jen Chen^1,2^, Feres Camargo Maluf^1^, Hannah Jarvis^1^, Zachary Burns^1^, Vicente Elorrieta^1^, Joseph Crivelli^1^, Thomas Chi^1^, Dean G Assimos^1^, Kyle D Wood^1^
^1^ Department of Urology, University of Alabama at Birmingham, Birmingham, Alabama, USA.
^2^ Department of Urology, Taipei Veterans General Hospital, Taipei City, Taiwan.

**Journal name:** World Journal of Urology

**Corresponding author:**
Kyle D Wood
[kwood@uabmc.edu](mailto:kwood@uabmc.edu)
Associate Professor
Department of Urology
University of Alabama at Birmingham

**Table S1** Stone characteristics and intra-operative characteristics

| Characteristics (55 renal units) |  |  |
| --- | --- | --- |
| Stone size (mm), median (Q1–Q3) | 24.0 (13.3–41.0) |  |
| Stone volume (mm^3^), median (Q1–Q3) | 1320.8 (582.7–2270.8) | |
| Stone numbers, median (Q1–Q3) | 1.0 (1.0–3.0) |  |
| Containing lower pole stones, n(%) | 34 (61.8) |  |
| Maximum stone HU, median (Q1–Q3) | 970.0 (625.0–1422.7) | |
|  |  |  |
| Characteristics (50 patients) |  | *p* value |
| Total operation time (minutes), mean±SD | 115.02±38.26 |  |
| For stone size >3cm (n=19) | 117.71±36.39 | 0.64 |
| For stone size ≤3cm (n=26) | 112.54±41.18 |  |
| Laser use, n(%) |  |  |
| Thulium fiber laser (Soltive, Olympus) | 45 (90) |  |
| Holmium laser (MOSES 2.0, Lumenis) | 5 (10) |  |
| CVAC type, n(%) |  |  |
| CVAC 1.0 | 17 (34) |  |
| CVAC 2.0 | 33 (66) |  |
| Use basket for stone removal, n(%) |  |  |
| Yes | 4 (8) |  |
| No | 46 (92) |  |
| Stone analysis results, most abundant component, n(%) |  |  |
| Calcium oxalate monohydrate | 18 (36.0) |  |
| Calcium oxalate dihydrate | 5 (10.0) |  |
| Calcium oxalate monohydrate +calcium oxalate dihydrate | 3 (6.0) |  |
| Carbonate apatite | 9 (18.0) |  |
| Brushite | 2 (4.0) |  |
| Uric acid* | 4 (8.0) |  |
| Struvite | 2 (4.0) |  |
| Ammonium urate | 4 (8.0) |  |
| No stone analysis data | 3 (6.0) |  |

SD, standard deviation; HU, Hounsfield Unit
* No pure uric acid stones were identified; all were of mixed composition containing calcium oxalate.
